# Surgical Management of Calciphylaxis Associated with Primary Hyperparathyroidism: A Case Report and Review of the Literature

**DOI:** 10.1155/2010/823210

**Published:** 2010-08-16

**Authors:** Jennifer Bishop, Eric Brown, Augusto Podesta, Cathrine Troy, Xiang (Eric) Dong

**Affiliations:** ^1^Department of Surgery, Stamford Hospital Columbia University, 30 Shelburne Road, Stamford, CT 06904, USA; ^2^Department of Nephrology, Stamford Hospital Columbia University, 30 Shelburne Road, Stamford, CT 06904, USA; ^3^Department of Pathology, Stamford Hospital Columbia University, 30 Shelburne Road, Stamford, CT 06904, USA; ^4^Department of Internal Medicine, Stamford Hospital Columbia University, 30 Shelburne Road, Stamford, CT 06904, USA

## Abstract

Calciphylaxis, or calcific uremic arteriolopathy, commonly affects people with end-stage renal disease and carries with it a high rate of morbidity and mortality. Here, we present the unusual case of a 56-year-old woman, with extensive medical problems, who developed calciphylaxis in the presence of primary hyperparathyroidism. Our patient initially presented with bilateral, exquisitely tender thigh lesions. The diagnosis of calciphylaxis was rendered histologically by extensive calcification of the subcutaneous blood vessels. Subsequent parathyroidectomy identified the presence of a hyperactive mediastinal parathyroid adenoma, weighing 0.62 grams. Postoperatively, the patient had normalization of hypercalcemia and parathyroid hormone levels, with subsequent healing of her thigh wounds. Currently, there have been sixteen cases described in the English literature, with only nine being offered a potentially therapeutic parathyroidectomy. It is contingent upon the vigilant physician to diagnose and properly manage this difficult yet treatable condition.

## 1. Introduction

Calciphylaxis is a rare disease entity first described by Bryant and White in 1898 to characterize a syndrome exemplified by vascular calcifications with cutaneous necrosis [1]. Attempts to shed light on its pathophysiology was undertaken by Selye et al. in 1962 using a rat model to induce calcinosis through a series of experimental steps [2, 3]. The condition was reproduced in nephrectomized rats as well as those challenged with sensitizers such as parathyroid hormone (PTH) and vitamin D [2, 3]. A series of sensitization events appears necessary to alter the calcium homeostasis prior to the induction of systemic calcinosis with another challenger agent [2, 3]. Therefore, patients can develop calciphylaxis even after renal transplantation or in the absence of end-stage renal disease such as hyperparathyroidism [4–6]. As the disease is multifactorial, many predisposing conditions contribute to the development of this serious disease, including morbid obesity, diabetes mellitus, malnutrition, liver disease, misuse of vitamin D and calcium-based phosphate binders, or coumadin intake [4–7]. 

The disease first gained notoriety after Gipstein et al. reported a series of 11 patients with calciphylaxis with concurrent end-stage renal disease [8]. Since his description, the incidence appears to be increasing within the last decade, afflicting 1%–4% of patients with end-stage renal disease [4, 6]. Attempts to characterize this patient population seem to concentrate on Caucasians with a female to male ratio of 3 to 1 [4, 6]. Although end-stage renal disease (ESRD) is the most likely culprit to result in disorders of calcium homeostasis, other conditions such as secondary hypercalcemia, hyperparathyroidism, sarcoidosis, or underlying connective tissue disease can also be potential etiologies to this disease entity [4]. The typical cutaneous manifestations of calciphylaxis, also seen in calcinosis cutis, comprise exquisitely tender and firm lesions with ninety percent located in the lower extremities [9]. The skin lesions of stellate purpura and livedo reticularis seen as a result of this disease arise from microthrombus formation within the small vessels [9, 10]. Both proximal and distal lesions can occur although the proximal lesions are usually located on the thighs and the lower abdomen. The proximal location helps to distinguish this disease from vascular causes of lower extremity skin necrosis.

## 2. Case Report

**Figure 1:**
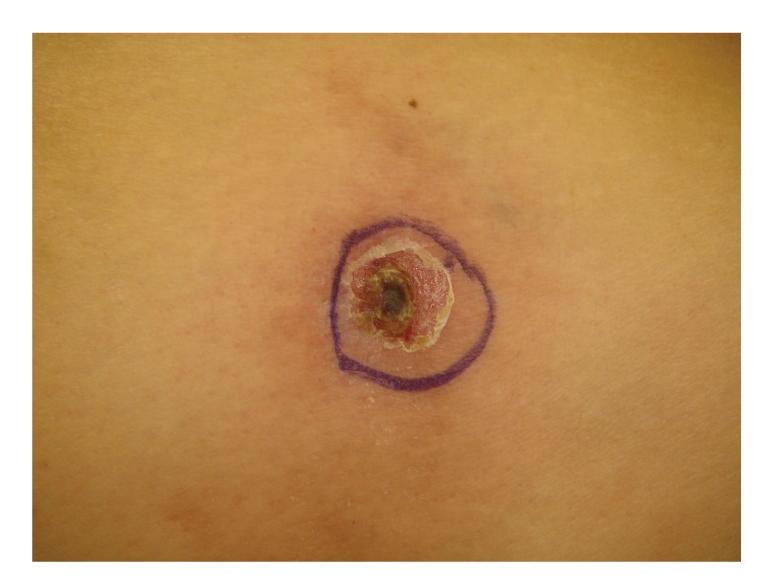
The appearance of our patient's bilateral thigh wounds showing firm cutaneous lesions that were exquisitely tender. The lesions are located in the proximal lower extremity on the lateral aspects of the thighs.

**Figure 2:**
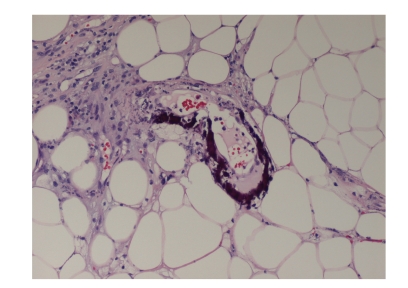
Skin biopsy with demonstration of vascular calcifications.

**Figure 3:**
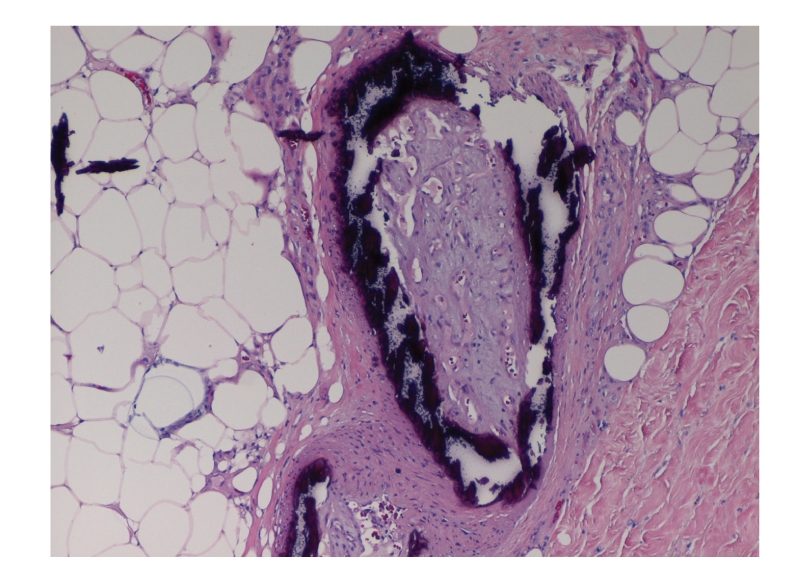
Skin biopsy with demonstration of cutaneous deposition of calcifications in addition to the vascular calcifications.

**Figure 4:**
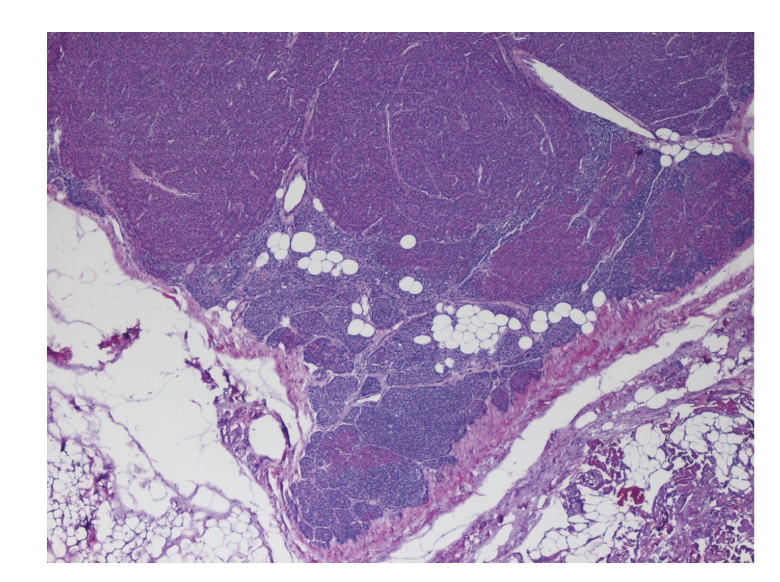
Evidence of primary hyperparathyroidism with hyperplastic cells within the parathyroid tissue. A paucity of adipose tissue is visible in the gland, suggesting the presence of a hyperplastic parathyroid.

This is the case of a 56-year-old Caucasian woman with extensive medical problems who initially presented with bilateral firm thigh lesions that are extremely painful and tender to touch (Figure 1). Her past medical history is complicated by type II diabetes, morbid obesity (BMI >35), and medically managed chronic renal insufficiency with a baseline creatinine level of 3.0 mg/dL. Interestingly, the patient had a history of poor wound healing following a hysterectomy in the past. Initial management consisted of a skin biopsy of these thigh lesions which led to the confirmation of calciphylaxis. Histologically, there were extensive calcifications within the subcutaneous tissue and small blood vessels (Figures 2 and 3). Surveillance chemistries revealed a preoperative intact PTH level of 737 pg/mL, with concurrent calcium level of 9.7 mg/dL, chloride level of 107 mmol/L, and phosphate level of 8.4 mg/dL. Preoperative vitamin D levels were examined and showed a normal total 25 hydroxy-vitamin D levels of 39 ng/mL. She then underwent elective parathyroidectomy with intraoperative PTH monitoring. Intraoperatively, the patient was found to have an enlarged nodular goiter as well as a 1.3-cm papillary carcinoma of the thyroid. Surprisingly, the patient was also found to have four normal parathyroids but an aberrantly located adenoma within the thymic tissue of the mediastinum, weighing 0.62 grams (Figure 4). Intraoperative PTH level remained elevated (>300 pg/mL) after excisional biopsy of three normally appearing parathyroid glands. Finally, the levels fell to 79 pg/mL after the removal of the enlarged adenoma, with a postoperative repeat value of 57 pg/mL. Postoperatively, the patient's thigh wound started to granulate with immediate improvement in her thigh pain.

## 3. Discussion

Historically, the first reported case of calciphylaxis from hyperparathyroidism was described by Ellis and Barr in 1951 [11]. The authors reported findings of a 29-year-old female with metastatic recurrent parathyroid carcinoma who had classic lesions of calciphylaxis during autopsy findings [12]. The recognition of this disease entity follows the early descriptions of Selye et al. in laboratory models of calciphylaxis [2, 3]. As the understanding of calciphylaxis became more widespread, reported cases of unusual causes of calciphylaxis also became more frequent [4, 5]. A search into the available data in the English literature identified a total of 15 other cases of patients with calciphylaxis secondary to primary hyperparathyroidism, with half of them being reported in the last decade [9, 11–22] (Table 1). The majority of patients succumbed to their disease due to the progressive nature of the disease [10]. However, a few patients did benefit from early parathyroidectomy and medical optimization [9, 12–18]. 

Initial reports of calciphylaxis secondary to nonrenal-related causes were usually a result of autopsy findings [11, 19, 20]. Bogdonoff and colleagues associated two autopsy findings of calciphylaxis with parathyroid adenomas in those patients in 1956 [19]. Both patients were males, which is much less common than females. Subsequently, Anderson et al. and Winkelman et al. also found parathyroid adenomas on autopsy findings of two sixty-two year old females and a sixty year old female in 1968 and 1970, respectively [9, 20]. With better understanding of the physiology of hyperparathyroidism and its effects on calcium homeostasis, surgeons are taking a more active role in treating this disease to decrease serum calcium levels in the hope of controlling the systemic manifestations associated with hypercalcemia. Winkelman et al. introduced parathyroidectomy as a management option in these patients and reported the first patient recovering from calciphylaxis caused by hyperparathyroidism in 1970 [9]. The second published report of a patient surviving after parathyroidectomy for hyperparathyroidism causing calciphylaxis was by Khafif et al. in 1989, on a 71-year-old female [12]. Despite the more aggressive approach undertaken recently, patients with calciphylaxis still have a guarded prognosis, with 80% succumbing to their disease secondary to recurrent skin infections [10, 23]. The disease can recur or their wounds can become progressive even without continued hypercalcemia. Even the role of parathyroidectomy is associated with much controversy [10]. Hafner et al. reported a survival advantage for patients who underwent parathyroidectomy in comparison to those who did not [23]. However, both Chan et al. and Roe et al. reported in their series that parathyroidectomy was not associated with a survival advantage in patients with end-stage renal disease [10, 24]. 

Calciphylaxis is not always a disease secondary to unopposed parathyroid hormone production from an aberrant gland. Buxtorf et al. presented an interesting case of a patient with hypercalcemia that was successfully managed with steroids and immunosuppression alone [4, 13]. There are also cases of calciphylaxis without association with renal failure or hyperparathyroidism reported in the literature [5]. This also correlates with the finding that patients with end-stage renal failure can have progressive calciphylaxis following renal transplantation and normalization of their parathyroid levels. It appears that once the disease process initiates following sensitization with hyperparathyroidism, there is difficulty in resetting the calcium homeostatic switch. However, parathyroidectomy may be the only option in these difficult cases especially with documented hyperparathyroidism, as evidenced by favorable results with early intervention recently [25]. 

This patient illustrates the various etiologies that can lead to calciphylaxis as well as the difficulty in managing this disease entity. Her disease presented with calciphylaxis secondary to primary hyperparathyroidism that improved with the use of parathyroidectomy. However, her chronic renal insufficiency and dysregulation of electrolyte balances initially suggested secondary hyperparathyroidism as the culprit for her calciphylaxis. Only during the surgery did we diagnose the presence of a single adenoma that resulted in the hyperparathyroidism. Vitamin D, though replete in this patient, can sometimes be a contributor to the development of hyperparathyroidism when deficient. In patients with calciphylaxis, early parathyroidectomy may still play a role in improving their cutaneous disease, especially in cases of hyperparathyroidism.

##  Author Contributions

All authors contributed to the writing and editing of this manuscript.

## Figures and Tables

**Table 1:** A list of documented calciphylaxis secondary to primary hyperparathyroidism in the world literature.

Case	Age/Sex	Pathology	Diagnosis	Intervention	Outcome	Reference
(1)	29 F	Carcinoma	Autopsy	Autopsy	Deceased	[11]
(2)	69 M	Adenoma	Autopsy	Autopsy	Deceased	[19]
(3)	44 M	Adenoma	Autopsy	Autopsy	Deceased	[19]
(4)	62 F	Adenoma (1.0 g)	Autopsy	Autopsy	Deceased	[20]
(5)	62 F	Adenoma (6.9 g)	Surgery	Surgery	Recovered	[9]
(6)	60 F	Carcinoma with metastases	Autopsy	Autopsy	Deceased	[9]
(7)	71 F	Adenoma	Surgery	Surgery	Recovered	[12]
(8)	46 F	Unknown	Leg ulcers	Steroids & Immunosupp.	Recovered	[13]
(9)	72 F	Adenoma	Leg ulcers	Conservative Management	Deceased	[21]
(10)	69 F	Adenoma (0.5 g)	Surgery	Surgery	Recovered	[14]
(11)	62 F	Hyperplasia	Leg ulcers	Surgery	Recovered	[17]
(12)	49 F	Adenoma (0.6 g)	Leg ulcers	Surgery	Recovered	[16]
(13)	76 F	Adenoma (0.33 g)	Leg ulcers	Surgery	Recovered	[18]
(14)	52 F	Adenoma	Surgery	Surgery	Recovered	[15]
(15)	52 F	Adenoma	Leg ulcers	Surgery	Deceased	[22]
(16)	56 F	Adenoma (0.62 g)	Leg ulcers	Surgery	Recovered	Bishop et al. 2010
